# Supplementary material for: ProteinShader: illustrative rendering of macromolecules
Source: BMC Struct Biol. 2009 Mar 30;9:19. doi: 10.1186/1472-6807-9-19 (PMC2672931; doi:10.1186/1472-6807-9-19)
Supplement: Additional file 1 — ProteinShader program without source code. This compressed file contains the complete ProteinShader program including associated libraries, but no source code. A README.txt file gives an overview of the ProteinShader distribution, and the index.html file in the help subdirectory has directions on getting started with the program as well as a set of tutorials. [file 1472-6807-9-19-S1.zip › ProteinShader-beta-0_9_4-binary/help/api/org/proteinshader/gui/listeners/menubar/DisplayMenuListenerFactory.html]

DisplayMenuListenerFactory (ProteinShader API)


|  |  |  |
| --- | --- | --- |
| **Overview** \| **Package** \| **Class** \| **Use** \| **Tree** \| **Deprecated** \| **Index** \| **Help** | |  |
| **PREV CLASS**   **NEXT CLASS** | **FRAMES**    **NO FRAMES**     **All Classes** |  |
| SUMMARY: NESTED \| FIELD \| CONSTR \| METHOD | DETAIL: FIELD \| CONSTR \| METHOD |  |


---


## org.proteinshader.gui.listeners.menubar Class DisplayMenuListenerFactory

```
java.lang.Object
  org.proteinshader.gui.listeners.menubar.DisplayMenuListenerFactory
```

---

``` public class DisplayMenuListenerFactory extends Object ```

Creates all event listeners needed by the DisplayMenu of the
MainMenuBar.

---

**Constructor Summary**

`DisplayMenuListenerFactory(Mediator mediator)`
  
          Constructs a DisplayMenuListenerFactory.


| **Method Summary** | |
| --- | --- |
| `ActionListener` | `createRadioButtonListener()`             Creates the action listener for all radio buttons in the DisplayMenu. |

**Methods inherited from class java.lang.Object**

`clone, equals, finalize, getClass, hashCode, notify, notifyAll, toString, wait, wait, wait`

**Constructor Detail**

### DisplayMenuListenerFactory

```
public DisplayMenuListenerFactory(Mediator mediator)
```

:   Constructs a DisplayMenuListenerFactory.

    **Parameters:**: `mediator` - the centralized Mediator object that listeners call on to accomplish their task.


**Method Detail**

### createRadioButtonListener

```
public ActionListener createRadioButtonListener()
```

:   Creates the action listener for all radio buttons in the
    DisplayMenu.

    :   **Returns:**: The action listener.


---


|  |  |  |
| --- | --- | --- |
| **Overview** \| **Package** \| **Class** \| **Use** \| **Tree** \| **Deprecated** \| **Index** \| **Help** | |  |
| **PREV CLASS**   **NEXT CLASS** | **FRAMES**    **NO FRAMES**     **All Classes** |  |
| SUMMARY: NESTED \| FIELD \| CONSTR \| METHOD | DETAIL: FIELD \| CONSTR \| METHOD |  |


---

# *Copyright © 2007-2008*
